# Iyengar-Yoga Compared to Exercise as a Therapeutic Intervention during (Neo)adjuvant Therapy in Women with Stage I–III Breast Cancer: Health-Related Quality of Life, Mindfulness, Spirituality, Life Satisfaction, and Cancer-Related Fatigue

**DOI:** 10.1155/2016/5931816

**Published:** 2016-02-25

**Authors:** Désirée Lötzke, Florian Wiedemann, Daniela Rodrigues Recchia, Thomas Ostermann, Daniel Sattler, Johannes Ettl, Marion Kiechle, Arndt Büssing

**Affiliations:** ^1^Institute of Integrative Medicine, University of Witten/Herdecke, Herdecke, 58313 Herdecke, Germany; ^2^Department of Psychology and Psychotherapy, University of Witten/Herdecke, Herdecke, 58448 Witten, Germany; ^3^Praxis Gynäkologie Arabella, 81925 Munich, Germany; ^4^Klinikum rechts der Isar, Technical University Munich, 81675 Munich, Germany

## Abstract

This study aims to test the effects of yoga on health-related quality of life, life satisfaction, cancer-related fatigue, mindfulness, and spirituality compared to conventional therapeutic exercises during (neo)adjuvant cytotoxic and endocrine therapy in women with breast cancer. In a randomized controlled trial 92 women with breast cancer undergoing oncological treatment were randomly enrolled for a yoga intervention (YI) (*n* = 45) or for a physical exercise intervention (PEI) (*n* = 47). Measurements were obtained before (*t*_0_) and after the intervention (*t*_1_) as well as 3 months after finishing intervention (*t*_2_) using standardized questionnaires. Life satisfaction and fatigue improved under PEI (*p* < 0.05) but not under YI (*t*_0_ to *t*_2_). Regarding quality of life (EORTC QLQ-C30) a direct effect (*t*_0_ to *t*_1_; *p* < 0.001) of YI was found on role and emotional functioning, while under PEI only emotional functioning improved. Significant improvements (*p* < 0.001) were observed at both *t*_1_ and *t*_2_ also for symptom scales in both groups: dyspnea, appetite loss, constipation, and diarrhea. There was no significant difference between therapies for none of the analyzed variables neither for *t*_1_ nor for *t*_2_. During chemotherapy, yoga was not seen as more helpful than conventional therapeutic exercises. This does not argue against its use in the recovery phase.

## 1. Background

The International Agency for Research on Cancer reported about “4.1 million new cancer cases […] in 2012 worldwide” [1]. The second most common form of cancer in the world is breast cancer. Operative interventions, adjuvant and neoadjuvant chemotherapy, radiotherapy, and endocrine therapy are the most common therapies for cancer in conventional medicine [2, 3]. However, cancer therapy has several attendant symptoms during and after therapy (e.g., a reduced physical fitness, fatigue, depression, and anxiety) [2, 4, 5]. In response to cancer therapy, a decrease in quality of life (QOL) is seen quite often. To reduce cancer therapies' side effects, the use of physical exercises is part of the treatment concept [6, 7].

Because of the complexity of attendant symptoms in cancer therapy, complementary approaches gain importance. Therapies referring to Mind-Body-Medicine (MBM) consider the whole person with all of his or her needs [4]. Most MBM interventions consider also patients' spirituality as relevant for disease management [4]. Spirituality may give an impulse for life reflection, or/and have positive impact on finding a meaning in life and/or for a reorientation in life [8, 9]. Yoga is among the most studied interventions of MBM and is assumed to “involve the union between mind, body, and spirit” (p. 2) [4]. The development of yoga began in the Indian culture [10]. Usually it is a combination of stretching exercises and various poses with a particular relevance of breathing and meditation [11].

The relevance of yoga in the scientific world was growing in the past years that is shown by the increase of scientific publications [10]. Previous research showed that yoga may have physiological as well as psychological effects for practitioners in a therapeutic context [11–13]. Different studies and systematic reviews confirm yoga's beneficial effects, for example, on fatigue, vigor, cortisol levels, emotional well-being, and QOL in breast cancer patients [14–19]. Yet, most studies were performed with cancer patients after their conventional anticancer treatment and compared yoga with passive control groups.

We thus aimed to test whether yoga has a more comprehensive effect on health-related QOL (HRQOL) issues, life satisfaction, cancer-related fatigue, mindfulness, and spirituality on patients with breast cancer during their therapy than conventional therapeutic exercises. This study uses an active control group to assess the unique contribution of yoga compared to other exercise interventions.

## 2. Methods

### 2.1. Patient Recruitment/Sample Size Calculation

In this anonym prospective, randomized trial data of breast cancer patients undergoing oncological treatment at the moment of intervention were analyzed.

Preliminary results in patients with shorter than 12 months of yoga practice found an Inner Congruence with Practices (ICPH) score of 64.9 ± 19.0 and 77.9 ± 12.6 in patients with 1–5 years of practice. Based on a statistical power of 1 − *β* = 0.8, a two-sided level of significance of *α* = 0.05, a conservative approximation of a minimal relevant difference of Δ_0_ = 10, and a pooled standard deviation of *s* = 16, a sample size of *n*_1_ = *n*_2_ = 42 was obtained. With an assumed dropout rate of 20% a total sample size of *n* = 102 (51 per group) seemed reasonable. Taking into account the short period of the intervention a sample size between *n* = 114 and *n* = 120 was finally considered as necessary to obtain the calculated sample size of 84 included patients.

After closing the database, 119 patients were included in the trial. Among them, 92 patients finished intervention so that their datasets were suited for statistical analysis. Patients were recruited from May 2011 to October 2014 in the Interdisciplinary Breast Center at the Klinikum rechts der Isar, Technical University of Munich (*n* = 102), and in a gynecological practice at Praxis Gynäkologie Arabella, Munich (*n* = 17).

Inclusion criteria were

- woman with stage I–III breast cancer,
- undergoing cytotoxic (neo)adjuvant or endocrine adjuvant therapy,
- signed informed consent.

Exclusion criteria were

- acute febrile or psychiatric diseases,
- regular practice of yoga or experience in practicing yoga.

Participants were randomized to one of the two intervention groups.

### 2.2. Intervention

Participants of the yoga intervention (YI) group received weekly a 60-minute session of regenerative Iyengar-Yoga over a period of 12 weeks at the intervention center “Yoga München GbR” in Munich. Iyengar-Yoga is a form of Hatha yoga developed by the Indian yoga teacher Iyengar (1980–2014). It refers to the traditional elements of yoga such as the positions (asanas) and breath control (pranayama) but is unique because of the use of “probs” (i.e., belts, blocks, ropes, and blankets) to help the practitioners to perform and keep the respective positions [20]. This makes it useful particularly for (weak) cancer patients as it uses in the beginning simple poses and thus minimizes exertions and decreases the risk of injury.

The comparison group received conventional physical exercise intervention (PEI) which consists of a 60-minute physical exercise session per week over a period of 12 weeks at the intervention center “Gesund. Reha rechts der Isar GmbH” in Munich.

Both groups were encouraged to perform home-based practices (YI and PEI) twice a week for 20 minutes supported by written instructions. All patients filled an exercise protocol for their own practice at home.

The study was approved in 2011 by the IRB of the Technical University of Munich (#3069/11).

### 2.3. Outcome Measures

Measurements were obtained before (*t*_0_) and after the 12-week intervention period (*t*_1_) as well as 3 months after finishing interventions (*t*_2_) using standardized questionnaires. In several cases, the intervention periods were longer as planned (due to cancer treatment regimens) and thus the 12 interventions refer to the respective individual time frame. Demographic information was obtained at baseline.

The following instruments were used.

*Health-Related Quality of Life.* To measure cancer patients' HRQOL, the cancer-specific EORTC QLQ-C30 (version 3.0) questionnaire was used. It consists of functional scales, symptom scales, a scale on the global health status, and single items according to cancer-related symptoms. Functional scales address physical functioning (i.e., strenuous activities, self-care, and long/short walk), role functioning (i.e., limited in work, limited in leisure), emotional functioning (i.e., depression, worry, tension, and irritability), cognitive functioning (i.e., concentration, memory trouble), and social functioning (i.e., family life, social activities). The symptom scales address nausea and vomiting, pain, dyspnea, sleep disturbance, appetite loss, constipation, diarrhea, and fatigue [21–23].

It uses a four-point Likert scale except the scales physical and role functioningwith dichotomous response choices and the scale concerning the global health status/quality of life scale on a seven-point scale [22]. For an easier interpretation the scales are converted and range from 0 to 100 [22, 23]. The symptoms scales are originally formulated negatively, but in order to better interpret the scores within the course of time, they were inverted as suggested by the EORTC Guideline [24]. Only the symptoms “fatigue” and “nausea and vomiting” were not inverted and in this case the greater the score, the higher the symptom.

*Life Satisfaction.* To measure life satisfaction, the Brief Multidimensional Life Satisfaction Scale (BMLSS) was applied. It has good internal consistency (Cronbach's alpha = .87) [25] and uses eight items addressing intrinsic, social, external, and perspective dimensions and three additional items on the health situation, abilities to deal with daily life activities, and the treatment success. Participants responded on a seven-point scale from dissatisfaction to satisfaction [25]. The BMLSS scores were referred to a 100% level (“delighted”).

*Fatigue.* Apart from EORTC QLQ-C30's fatigue symptom scale, we also used the 15-item Cancer Fatigue Scale (CFS-D) [26]. It has a very good reliability coefficient (alpha = .94) and differentiates three dimensions, that is, affective, cognitive, and physical fatigue [26].

The instrument uses a five-point Likert scale with a range from 0 (not at all) to 4 (extraordinary).

*Mindfulness.* To quantify mindfulness, we used the 14-item Freiburg Mindfulness Inventory (FMI) (Freiburger Achtsamkeits-Inventar) with good internal consistency (Cronbach's alpha = .86) [27]. Descriptive statements are, for example, “I am open to the experience of the present moment,” “I sense my body, whether eating, cooking, cleaning, or talking,” and “I see my mistakes and difficulties without judging them” [27]. The scales have one common factor and correlated strongly with a person's self-awareness. Nevertheless, it covers the topics of acceptance (related to the nonjudgmental acceptance of the situation) and presence (related to the experience of the moment and a cognitive reflection of all actions). Response categories are rarely, occasionally, fairly often, and almost always [27, 28].

*Spiritual Attitudes and Coping with Illness.* To investigate whether or not patients rely on spirituality as a resource to cope with illness, we used the SpREUK questionnaire [29]. In its 15-item version it differentiates* search *for support/access to spirituality/religiosity,* trust* in higher guidance/source, and* reflection*: positive interpretation of disease [30]. The search scales deal with patients' intention to find or have access to a spiritual/religious resource which may be beneficial to cope with illness and interest in spiritual/religious issues (insight and renewed interest). The trust scale is a measure of intrinsic religiosity dealing with patients' conviction to be connected with a higher source which carries through and to be sheltered and guided by this source, whatever may happen. The reflection (positive interpretation of disease) scale deals with cognitive reappraisal because of illness and subsequent attempts to change (i.e., reflecting on what is essential in life, hint to change life, chance for development, illness that has meaning, etc.). The internal consistency coefficients (Cronbach's alpha) of the three subscales range from .86 to .91 [30].

Items were scored on a five-point Likert scale ranging from disagreement to agreement.

### 2.4. Statistical Analysis

All data analyses were performed with SPSS 22.0.

To test whether the two groups differ at baseline in terms of the sociodemographic data and the outcomes of interest, Pearson's Chi^2^ test was used for categorical variables and for continuous variables either the *t*-test for normal distributed data or the Mann-Whitney test for nonnormal distributed data, respectively.

To compare data during the course of time (*t*_0_, *t*_1_, and *t*_2_), the ANOVA for repeated measures was used when the data assumed normality. The nonparametric test of Friedman was used when no normality on the data was observed.

The nonparametrical test of Kruskal Wallis was used as a nonparametrical ANOVA. A nonparametrical measure of concordance between the time points was also reported to better understand the relationship between these measurements and also to not rely only on *p* values. Kendall's coefficient of concordance “*W*” gives the proportion of agreement in the ranking of responses between measurements. Here, *W* = 0 means no agreement, indicating that ranks are completely random, whereas *W* = 1 indicates complete consistency between measurements. Thus, *W* gives a scaled measure of the effect size of consistency in ranks between measurements [31]: higher *W* values indicate stronger concordance.

To handle missing data, the multiple imputation MI method was used [32]. Although the last observation carried forward (LOCF) method is often applied for clinical studies, the National Research Council advised in 2010 [33] not to use this method because it leads to biased estimates. In our study, almost 55% of the *t*_2_ data are missing, and therefore all statistical analyses were performed on an intention-to-treat basis.

The significance level considered in the analysis was 5%, *α* = 0.05 when the data without repeated measures was used, and after a Bonferroni correction for repeated measures (using three time measurements) the significance level was set at *α* = 0.017, when using only two time measurements *α* = 0.025.

For this analysis, the main outcome variable was patients' HRQOL (EORTC QLQ-C30).

## 3. Results

Patients were randomized to either the YI (*n* = 45) or the PEI group (*n* = 47). Three patients dropped out at *t*_1_ and finally 54 patients at *t*_2_ (59%). Reasons could not be documented clearly. At *t*_1_, we had data of 43 YI patients (2 dropouts) and 46 from PEI (1 dropout); at *t*_2_ we had data of 16 YI patients (29 dropouts) and 22 from PEI (25 dropouts). Thus, the missing data were imputed using Rubin's multiple imputation MI method.

### 3.1. Baseline Data

Sociodemographic data of enrolled patients at baseline are displayed in Table 1. A significant difference in the sociodemographic data between groups was found only for the educational level. There were no significant differences between groups for the outcome variables (Table 2). We were unable to get further data on tumor grading, receptor status, and so forth. However, the treatment schemata did not significantly differ between both groups, yet there were somewhat more patients with endocrine therapy in the PEI group and more with chemotherapy and radiation in the YI group.

### 3.2. Direct Effects of Intervention (*t*_0_ to *t*_1_)

#### 3.2.1. EORTC's Functional Scales

PEI patients significantly improved on emotional functioning (*p* < 0.001, *W* = 0.296), while YI patients improved on role functioning (*p* = 0.013, *W* = 0.139) and emotional functioning (*p* = 0.018, *W* = 0.093). There was no significant difference between both groups regarding these scales (Table 3).

#### 3.2.2. EORTC's Symptom Scales

PEI patients improved significantly (*p* < 0.001) on dyspnea (*W* = 0.277), appetite loss (*W* = 0.539), constipation (*W* = 0.594), and diarrhea (*W* = 0.893). Similarly, YI patients improved on dyspnea (*W* = 0.517), appetite loss (*W* = 0.679), constipation (*W* = 0.888), and diarrhea (*W* = 0.909), too. For “fatigue,” “nausea and vomiting,” “pain,” and “sleep disturbance” there were no significant changes over time. There were no significant differences between both intervention groups.

#### 3.2.3. Life Satisfaction

Life satisfaction did not improve in YI (*p* = 0.366, *W* = 0.018) or in PEI group (*p* = 0.366, *W* = 0.017). Both did not differ significantly.

#### 3.2.4. Cancer-Related Fatigue

Both interventions did not significantly improve CRF (YI's *p* = 0.763, *W* = 0.066; PEI's *p* = 0.180, *W* = 0.038). Both groups did not significantly differ.

#### 3.2.5. Spirituality and Mindfulness

For patients in the YI group, neither search, trust, nor mindfulness improved significantly, while reflection did improve (*p* = 0.002, *W* = 0.217). However, search (*p* = 0.009, *W* = 0.143) and trust (*p* = 0.024, *W* = 0.109) significantly improved in PEI. Both groups did not significantly differ for these variables.

### 3.3. End of Intervention (*t*_0_, *t*_1_ to *t*_2_)

#### 3.3.1. EORTC's Functional Scales

Statistically significant results were found for most functional scales of the EORTC which indicates the spontaneous recovery of patients' QOL after chemotherapy/radiation.

Patients of both groups improved significantly on global health, role, and social functioning. Yet, both groups did not significantly differ from each other for these variables (Table 3).

#### 3.3.2. EORTC's Symptom Scales

YI and PEI patients improved on fatigue, dyspnea, appetite loss, constipation, and diarrhea. For “nausea and vomiting” and “pain” there were no significant changes over time. Both groups did not significantly differ.

#### 3.3.3. Life Satisfaction

Life satisfaction was quite high at baseline for YI patients, and thus no statistical improvement was observed (*p* = 0.03, *W* = 0.078), while PEI patients did significantly improve (*p* < 0.001, *W* = 0.165). Yet, there were no significant differences between both interventions.

#### 3.3.4. Cancer-Related Fatigue

For YI we observed only a remarkable trend (*p* = 0.052, *W* = 0.066), while in PEI group a significant improvement was found (*p* = 0.013, *W* = 0.092). There was no significant difference between both groups.

#### 3.3.5. Spirituality and Mindfulness

There were no significant differences between interventions for the SpREUK subscales, but for mindfulness (*p* = 0.034).

## 4. Discussion

This study aimed to test the effectiveness of yoga for women with stage I–III breast cancer during (neo)adjuvant cytotoxic and endocrine therapy on HRQOL, life satisfaction, cancer-related fatigue, mindfulness, and spirituality compared to conventional physical exercise.

Analyzing both therapies first separately, we saw no significant differences between YI and PEI regarding life satisfaction, fatigue, and mindfulness for the first comparison (*t*_0_ − *t*_1_) or for the second comparison (*t*_0_ − *t*_2_). Patients in PEI group showed improvements on life satisfaction and on cancer-related fatigue at the second follow–up assessment, but not directly after the intervention. In contrast to our findings, a number of studies reported significant reduction on fatigue after YI in women with breast cancer [14, 15, 34–36]. This might be explained by the fact that the effectiveness of yoga depends on the treatment status (patients within the treatment or cancer survivors) [20].

A possible mechanism by which yoga could have positive effects on different aspects of QOL might be its spiritual aspect [37]. In this study, a significant improvement on patients' ability to reflect their life concerns was found in the YI group, and, however, an increase on spiritual search and religious trust scores for PEI patients at  *t*_1_. Nevertheless, no significant differences between both therapies were found on these dimensions. A small number of previous studies assessed issues of spirituality after a YI in cancer patients. Moadel et al., for example, reported significant improvements on spiritual well-being after a 12-week YI in multiethnic breast cancer patients [38]. In contrast Danhauer et al. found no significant changes for spirituality after 10 weeks of yoga in ovarian or breast cancer patients [34]. Again, these differences might be mainly due to the differences in the phases of observation. Nevertheless, we have no rationale why particularly the patients in the PEI group showed changes in trust and search although these topics are not specifically addressed in the PEI group.

EORTC QLQ-C30's functioning scales significantly improved only on role and emotional functioning after YI and on emotional functioning in PEI group at *t*_1_. On all other scales, no significant differences were found for the pre- and postmeasures. Comparing these interventions, no statistical difference could be found. Vadiraja et al. also assessed QOL using the EORTC QLQ-C30 questionnaire with focus on the functional scales in breast cancer patients undergoing adjuvant radiotherapy. In contrast to our findings they reported significant effects on emotional and cognitive functioning, but no effects on role functioning [37].

For the symptom scales of the EORTC QLQ-C30, dyspnea, appetite loss, constipation, and diarrhea, there were significant improvements over time for both therapies, at both *t*_1_ and *t*_2_. This is in line with the results of Rao et al. who found reduced symptom distress in (breast) cancer patients after YI [39]. The most common symptoms from chemotherapy, “nausea and vomiting” and “pain,” could not be improved in this study.

The present findings are in line with the results of previous research on QOL in breast cancer patients after YI which also found positive effects on this patient relevant outcome parameter [17, 18]. Levine and Balk summarized in their review on yoga and QOL in breast cancer patients that there are beneficial effects, for example, better coping with side effects, positive effects on emotional and cognitive functioning, reduced anxiety, and depression symptoms. However they emphasized that it remains unclear which mechanisms are responsible for its effectiveness and which components of yoga are the most important ones to increase QOL [40].

Because of the fact that cancer patients often perceive barriers to be physically active [41], it seems necessary to find attractive and effective supportive treatments in the therapy of breast cancer patients to improve HRQOL. In this study, positive results were found between the baseline assessment and the first follow-up assessment after 12 weeks of therapy for relevant outcome parameters; this indicates yoga as an effective intervention under specific conditions. However, the greatest improvements were found between *t*_1_ and *t*_2_ what is not surprising because most of the patients already have completed chemotherapy at that time. A certain proportion of improvements in QOL would thus be explained due to (spontaneous) recovery effects.

We see a need for further research for more specific information on the effects of YI during chemotherapy. Stan et al. summarized that because of “the increasing interest in yoga for cancer survivors and medical institutions, it appears that yoga is establishing itself into the mainstream management and treatment of cancer survivors” (p. 11) [4]. But the authors point out that previous studies have to be interpreted with caution because of small sample sizes and study limitations [4]. This study is a further step to close existing research gaps in this area as it was applied during patients' chemotherapy phase instead of monitoring only the effects in their phases of convalescence.

To integrate yoga as supportive treatment during therapy in women with breast cancer, it will be necessary to spend more research activities on safety aspects [10]. A further challenge to integrate yoga in the therapeutic context and to improve the quality of interventions, standardized and improved curricula for yoga teachers are needed [10].

## 5. Limitations

Because of the high dropout rate between the first follow-up assessment and *t*_2_ and therefore the large number of imputed data pieces, the results on the basis of these data pieces have to be interpreted with caution. The high dropout rate may reflect a low acceptance for supportive interventions during cancer treatment. Possibly an additional intervention during cancer treatment is too exhausting for breast cancer patients.

Another limitation could be that patients did not participate consistently and the sample size was not large enough to compensate for the dropouts. The number of lessons varied from 5 to 12, and the lapse of time, in between the patients performed, varied from 6 weeks to 25 weeks. This variation was not considered in the analysis. Maybe in some cases the frequency of training was just too poor to produce observable effects. Hence further research should also focus on extent and frequency of YI to learn more about appropriate training designs for patients undergoing chemotherapy.

## 6. Conclusion

The findings indicate that yoga may have beneficial effects on QOL issues in women with stage I–III breast cancer during (neo)adjuvant cytotoxic and endocrine therapy. Patients' life satisfaction and cancer-related fatigue improved in both intervention groups, yet without significant between-group effects. Further studies on the specific effects of yoga during the phases of chemotherapy and radiotherapy are needed. So far, yoga is not better than conventional physical exercises. It might be that yoga styles and their components may vary in their effectiveness on outcomes in breast cancer patients. Thus, future research should therefore examine whether other styles create differing results.

## Figures and Tables

**Table 1:** Sociodemographic data of enrolled patients with breast cancer (baseline data).

Variables	YI	PEI	*p* value
	Frequency or means		
Intervention	45	47	
Age	51.0 ± 11.0	51.4 ± 11.1	0.882
Family status			
Married	23	29	0.531
Living with partner	8	8	
Divorced	3	3	
Single	8	3	
Widowed	3	2	
Education level			
Secondary (Hauptschule)	0	5	0.049^*∗*^
Junior high school (Realschule)	14	20	
High school (gymnasium)	24	18	
Other	6	3	
Therapy			
Neoadjuvant chemotherapy	7	7	0.120
Adjuvant chemotherapy	19	29	
Endocrine therapy	1	3	
Radiation	0	1	
Endocrine therapy + radiation	12	3	
Chemotherapy + radiation	2	2	
Chemotherapy + radiation + endocrine therapy	4	2	

^*∗*^Significant at a significance level of 5%.

**Table 2:** Significant differences of outcome variables between both groups at baseline.

Variables	*p* value^*∗*^
Quality of life: EORTC's functional scales	
Global health score	0.917
Physical functioning	0.190
Role functioning	0.753
Emotional functioning	0.060
Cognitive functioning	0.164
Social functioning	0.697

Quality of life: EORTC's symptom scales	
Nausea and vomiting	0.923
Pain	0.721
Dyspnea	0.347
Sleep disturbance	0.544
Appetite loss	0.360
Constipation	0.225
Diarrhea	0.487
Fatigue	0.434

Other health-related variables	
Cancer-related fatigue (CFS-D)	0.141
Life satisfaction (BMLSS)	0.480

Spirituality	
Mindfulness (FMI)	0.179
Spiritual search (SpREUK)	0.214
Religious trust (SpREUK)	0.811
Reflection (SpREUK)	0.903

^*∗*^Significant at a significance level of 5%

(*t*-test for normal distributed variables was used for CFS-D, FMI, BMLSS, and EORTC's global health; all other variables were nonnormally distributed and thus the Mann-Whitney test was used).

**Table 3:** Mean values for variables per time measurement (ITT dataset).

Variables	YI					PEI					YI versus PEI	
	*t*_0_	*t*_1_	*t*_2_	*p* value *t*_0_ to *t*_1_	*p* value *t*_0_, *t*_1_, *t*_2_	*t*_0_	*t*_1_	*t*_2_	*p* value *t*_0_ to *t*_1_	*p* value *t*_0_, *t*_1_, *t*_2_	*t*_1_	*t*_2_
	Mean values (±SD)					Mean values (±SD)					*p* value	
Health-related quality of life: EORTC's functional scales												
Global health	57.39 (±20.50)	60.37 (±17.95)	75.00 (±13.05)	0.862	** <0.0001**	56.91 (±22.40)	58.33 (±21.13)	73.04 (±16.04)	0.739	** <0.0001**	0.611	0.553
Physical functioning	113.33 (±14.87)	113.33 (±18.85)	114.96 (±12.72)	0.182	0.767	108.00 (±17.79)	108.08 (±21.66)	112.34 (±13.96)	0.612	0.170	0.258	0.309
Role functioning	82.19 (±23.40)	90.00 (±22.01)	104.81 (±19.98)	** 0.013**	**<0.0001**	79.62 (±29.27)	90.42 (±38.50)	109.57 (±21.63)	0.106	**<0.0001**	0.625	0.161
Emotional functioning	92.05 (±23.07)	94.25 (±22.24)	103.51 (±15.53)	** 0.018**	0.018	83.69 (±25.02)	85.17 (±26.42)	104.78 (±19.71)	**<0.0001**	** <0.0001**	0.168	0.032
Cognitive functioning	108.91 (±21.00)	102.22 (±21.78)	111.48 (±17.70)	0.144	0.134	100.72 (±26.28)	98.22 (±28.28)	106.02 (±22.90)	0.602	** 0.007**	0.601	0.277
Social functioning	89.77 (±26.46)	86.66 (±28.99)	102.59 (±17.02)	0.739	** 0.011**	91.67 (±28.05)	91.84 (±32.20)	105.31 (±20.29)	0.732	** 0.008**	0.423	0.302

Health-related quality of life: EORTC's symptom scales/items												
Fatigue^*∗∗*^	15.90 (22.01)	18.51 (±26.90)	4.19 (±17.12)	0.732	** 0.002**	20.53 (±26.60)	17.49 (±30.71)	2.36 (±19.09)	0.612	** 0.001**	0.906	0.408
Nausea and vomiting^*∗∗*^	−21.59 (±18.53)	−22.22 (±17.76)	−22.59 (±14.71)	0.808	0.924	−20.21 (±23.55)	−24.11 (±18.32)	−26.59 (±12.36)	0.197	0.457	0.571	0.157
Pain	1.13 (±29.28)	2.96 (±30.41)	−4.81 (±20.90)	0.853	0.036	−0.37 (±30.46)	1.41 (±33.48)	−0.35 (±23.17)	0.433	0.795	0.684	0.347
Dyspnea	−9.85 (±24.46)	34.07 (±28.85)	42.22 (±25.02)	**<0.0001**	** <0.0001**	−2.89 (±30.49)	32.62 (±34.39)	43.26 (±29.41)	** <0.0001**	**<0.0001**	0.948	0.589
Sleep disturbance	18.18 (±29.16)	22.96 (±30.83)	24.22 (±32.87)	0.480	0.030	14.18 (33.86)	16.31 (±33.95)	29.07 (±32.31)	0.622	0.043	0.328	0.034
Appetite loss	−14.39 (±29.11)	42.96 (±31.48)	57.77 (±16.51)	**<0.0001**	**<0.0001**	−8.51 (±32.20)	47.51 (±30.88)	60.99 (±12.66)	**<0.0001**	**<0.0001**	0.369	0.359
Constipation	−18.93 (±20.83)	54.07 (±22.79)	57.03 (±15.27)	**<0.0001**	** <0.0001**	−8.51 (±32.20)	48.93 (±26.78)	59.57 (±15.44)	**<0.0001**	** <0.0001**	0.369	0.308
Diarrhea	−21.21 (±26.01)	49.62 (±26.22)	57.03 (±16.85)	**<0.0001**	** <0.0001**	−25.36 (±20.10)	52.48 (±23.81)	55.31 (±19.99)	**<0.0001**	** <0.0001**	0.636	0.825

Other health-related variables												
Cancer-related fatigue (CFS-D)	19.87 (±10.09)	21.04 (±9.91)	17.60 (±6.05)	0.763	0.052	23.30 (±10.51)	24.32 (±10.63)	18.59 (±7.08)	0.180	** 0.013**	0.168	0.431
Life satisfaction (BMLSS)	71.16 (±16.62)	72.79 (±15.74)	77.63 (±11.13)	0.366	0.029	68.68 (±16.23)	69.12 (±13.83)	77.18 (±9.52)	0.366	** <0.0001**	0.085	0.087

Spirituality												
Mindfulness (FMI)	1.93 (±0.50)	1.96 (±0.50)	1.98 (±0.31)	0.639	0.364	1.78 (±0.47)	1.80 (±0.45)	1.90 (±0.28)	0.881	0.214	0.041	0.034
Spiritual search (SpREUK)	42.20 (±28.66)	45.33 (±26.57)	41.66 (18.37)	0.250	0.541	34.68 (±25.28)	40.00 (±28.85)	40.10 (±22.10)	** 0.009**	0.116	0.347	0.953
Religious trust (SpREUK)	50.69 (±25.78)	54.22 (±23.95)	55.33 (±19.49)	0.040	0.246	51.81 (±34.45)	55.42 (±33.61)	49.47 (±26.52)	** 0.024**	0.043	0.736	0.245
Reflection (SpREUK)	62.90 (±26.66)	66.22 (±26.88)	62.33 (±19.17)	** 0.002**	0.055	64.36 (±22.08)	66.38 (±20.34)	64.46 (±14.93)	0.150	0.514	0.539	0.574

^*∗∗*^Higher scores = higher symptoms.
